# Differential Alternating Current Field Measurement with Deep Learning for Crack Detection and Evaluation

**DOI:** 10.3390/mi16030318

**Published:** 2025-03-10

**Authors:** Chenxu Fan, Zhenhu Jin, Jiamin Chen

**Affiliations:** 1 State Key Laboratory of Transducer Technology, Aerospace Information Research Institute, Chinese Academy of Sciences, Beijing 100190, China; fanchenxu20@mails.ucas.ac.cn; 2 School of Electronic, Electrical and Communication Engineering, University of Chinese Academy of Sciences, Beijing 100049, China; 3 College of Materials Sciences and Opto-Electronic Technology, University of Chinese Academy of Sciences, Beijing 100049, China

**Keywords:** alternating current field measurement, tunnel magnetoresistance, deep learning, nondestructive testing

## Abstract

This paper introduces a novel differential TMR-ACFM probe integrated with deep learning for crack detection and evaluation. The differential design effectively mitigates the lift-off effect and external noise, thereby enhancing detection performance without increasing costs. A miniature TMR was designed and fabricated for the probe. Two TMR units were integrated in an area of 175 × 200 microns, and two dies formed the differential structure of the Wheatstone bridge. Experimental results indicate that, in comparison to conventional probes, the quality factor of the differential probe is improved by more than an order of magnitude, and the signal-to-noise ratio is enhanced by over 3 dB. Additionally, a CNN + CBAM network is developed and trained on experimental data to achieve high-precision evaluation of crack dimensions. For cracks measuring 10–30 mm in length, 2–6 mm in depth, and 0.25–1.25 mm in width, the relative errors in the predicted dimensions are 0.201%, 0.709%, and 7.224%, respectively. These results underscore the significant potential of the proposed approach for quantitative crack detection.

## 1. Introduction

Alternating current field measurement (ACFM) is an electromagnetic nondestructive testing (NDT) technique that has been extensively employed for detecting metal cracks, particularly within the realm of structural health monitoring across industries such as aviation, shipping, and petroleum [1,2]. Its non-contact operation and robust performance under harsh environmental conditions underscore its significant potential for practical applications [3].

Developed from the alternating voltage drop method [4], ACFM utilizes excitation sources—such as coils and yokes—to generate an alternating magnetic field at a specified distance from the surface of the test specimen. This excitation induces a uniformly distributed current along the surface and in the near-surface regions. The resulting secondary magnetic field, generated by this induced current, is detected by magnetic sensors, including coils [5], giant magnetoresistance (GMR) [6], or tunnel magnetoresistance (TMR) [7,8] elements. Among these, TMR sensors offer distinct advantages—namely, a wide frequency bandwidth, compact size, high sensitivity, and ease of integration—making them the preferred choice for ACFM applications [9,10]. When defects such as cracks are present, the current distribution is disrupted, leading to measurable changes in the secondary magnetic field. Analysis of these variations facilitates not only the detection of defects but also the estimation of their characteristic parameters.

Despite the advances achieved with ACFM, certain challenges persist, notably the false detections induced by the lift-off effect and noise from external magnetic fields. To address the lift-off effect, various compensation strategies have been proposed. For example, Yuan et al. analyzed the influence of lift-off distance on ACFM measurements and introduced a compensation method that enables accurate crack size detection at any fixed lift-off distance [11]. Similarly, Zhao et al. identified a lift-off point of intersection (LOI) feature in the raw ACFM data that could be exploited to suppress associated noise [12]. However, these approaches have limitations: the former requires measurement of magnetic field components along both the x and z axes, while the latter may lead to signal distortion and a consequent reduction in detection sensitivity. Additionally, other researchers have explored the use of external magnetic shielding materials or signal processing algorithms to mitigate external noise. Liu et al. employed ferrite cores to shield against magnetic interference [13], and Sun et al. applied a denoising algorithm to reduce noise in ACFM signals [14]. Although effective to some extent, these methods necessitate additional hardware or complex signal processing systems.

In recent years, machine learning and deep learning have significantly improved defect evaluation based on ACFM. In 2021, Li Wei et al. applied a Convolutional Neural Network (CNN) to ACFM for defect identification [15]. In 2022, Zhao Jianming et al. developed an end-to-end physics-informed neural network for defect identification and 3D reconstruction using 2D magnetic field map data from ACFM [16]. In 2024, Yuan Xin’an et al. introduced a dual-gradient fusion imaging method with a Conditional Denoising Diffusion Model (CDDM) for defect reconstruction [17]. Deep learning has greatly expanded the information obtainable from ACFM, evolving from basic size measurements to detailed 3D reconstructions. The main challenges now focus on improving evaluation accuracy and model versatility.

To address these challenges, a differential TMR-ACFM probe design is proposed. This design inherently suppresses signal variations resulting from changes in lift-off distance and counteracts the influence of external noise without the need for supplementary external devices or signal processing modules. Differential designs have been successfully implemented in other nondestructive testing techniques, such as eddy current testing [18] and magnetic flux leakage testing [19], demonstrating their potential to address issues related to lift-off effects and magnetic interference. In this work, a novel differential design approach to ACFM is introduced and validated through comprehensive theoretical analysis and experimental verification. Furthermore, leveraging the differential TMR-ACFM system, a deep learning framework is developed that integrates a Convolutional Neural Network (CNN) with a Convolutional Block Attention Module (CBAM) to enhance crack detection performance.

## 2. Principle and Design

### 2.1. Physical Principle

**Figure 1:**
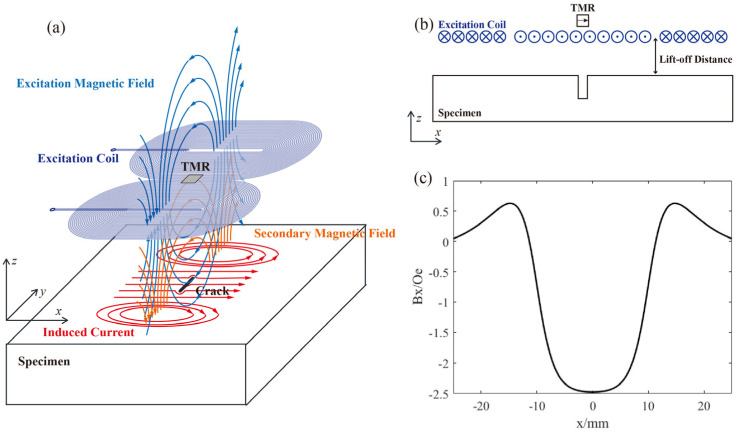
(**a**) Schematic diagram of the ACFM principle; (**b**) schematic diagram of the TMR-ACFM; (**c**) the ideal ACFM output curve.

The ACFM probe mainly consists of an excitation source and a sensitive unit [3,20]. A typical ACFM configuration uses a planar coil as the excitation source and a single TMR at its center as the sensitive unit. The basic detection principle is shown in Figure 1a. When an alternating current is passed through the coil, an alternating excitation magnetic field is generated around it. Under the excitation of the excitation magnetic field, an induced electric field is generated on and near the surface of the metal. The induced electric field simultaneously generates a secondary magnetic field in the surrounding space. If there are defects on and near the surface of the specimen, the flow direction and density of the induced current will be disturbed, which in turn causes changes in the magnetic field signal detected by the magnetic sensitive unit.

Among various nondestructive testing (NDT) techniques for metal defect detection, ultrasonic testing (UT) is insensitive to surface and near-surface defects, machine vision methods cannot detect defects beneath coatings, radiographic testing (RT) is costly and lacks portability, and magnetic flux leakage (MFL) is limited to ferromagnetic metals. Meanwhile, eddy current testing (ECT) falls short of alternating current field measurement (ACFM) in terms of precision. Given these limitations, ACFM plays a crucial role in detecting surface and near-surface defects in metals. This work employs TMR as the sensitive element for ACFM. TMR sensors are known for their high sensitivity, low power consumption, small size, and high integration, allowing for flexible probe design according to different detection scenarios. They also exhibit excellent temperature stability, reliability, and long lifespan, making them suitable for use in complex conditions. Therefore, ACFM probes based on TMR show broad application prospects in the industrial inspection field.

ACFM is characterized by a uniform current distribution in the detection area and a stable output signal, which is conducive to the inversion of defect information. The magnetic field is a vector signal, and TMR is usually uniaxially sensitive. A common configuration is to place the sensitive axis of TMR in the x-axis or z-axis direction. In the former case, there are signal fluctuations throughout the defect area, while in the latter case, there are only signal fluctuations at the edges of the defect. This paper only discusses the case where the sensitive direction of TMR is the x-axis, as shown in Figure 1b. Ideally, the magnetic field change curve detected by TMR is as shown in Figure 1c, with peaks at the edges of the defect and valleys in the defect area. However, in the actual detection process, the magnetic field detected by TMR consists of three parts:

(1) $$B_{x}=B_{Coil}+B_{Noise}+B_{Secondary}\left(d_{lo},J_{Induced}\right).$$

Specifically, they are the excitation magnetic field $B_{Coil}$ generated by the excitation coil, the environmental magnetic field noise $B_{Noise}$, and the secondary magnetic field $B_{Secondary}$. During the ACFM detection process, the factors that negatively affect the detection results are mainly reflected in the following two aspects: First, the dynamic components of the excitation magnetic field $B_{Coil}$ and the environmental magnetic field noise $B_{Noise}$ will directly cause fluctuations in the detection signal, thereby leading to misdetection. Their static components, on the other hand, will affect the reference value of the output signal, ultimately having an adverse effect on the detection sensitivity. Second, during actual detection, changes in the lift-off distance $d_{lo}$ are inevitable. Since the lift-off distance is related to the reference value of the signal, its variation will cause the signal to fluctuate, and such fluctuations will also lead to misdetection issues.

**Figure 2:**
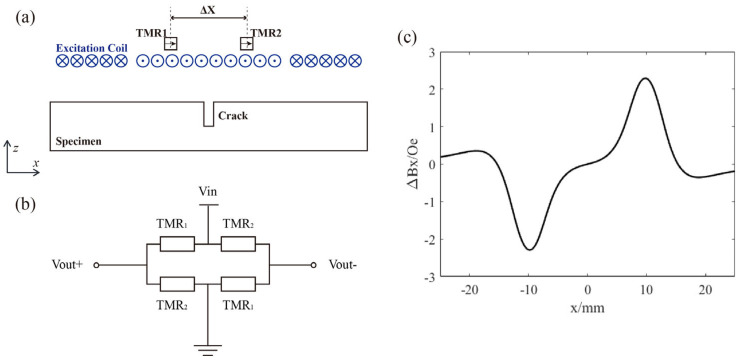
(**a**) Schematic diagram of the differential TMR-ACFM; (**b**) schematic of the Wheatstone bridge circuit; (**c**) ideal differential ACFM output curve.

To address these issues, a differentially designed ACFM system is proposed. As shown in Figure 2a, two sets of TMR sensors are symmetrically placed within the detection plane along the center of the excitation coil at ∆X. Each set consists of two TMR units with overlapping sensitive regions, and all four TMR units share the same sensitivity direction. These four TMR units form a Wheatstone bridge for signal output, as illustrated in Figure 2b.

The resistance of each TMR unit is given by the following:

(2) $$R_{TMR}=R_{0}+S_{R}B_{x},$$

where $R_{0}$ is the resistance in the absence of a magnetic field, and $S_{R}$ represents the magnetoresistive sensitivity of the TMR sensor. The output of the Wheatstone bridge is given by the following:

(3) $$Output=V_{out+}-V_{out-}=\frac{R_{TMR1}-R_{TMR2}}{R_{TMR1}+R_{TMR2}}V_{in}\approx \frac{S_{R}V_{in}}{2R_{0}}\Delta B_{x},$$

where $V_{out+}$ and $V_{out-}$ are the voltages at both ends of the Wheatstone bridge, $V_{in}$ is the supply voltage, $R_{TMR1}$ and $R_{TMR2}$ are the resistances of the two sets of TMR sensors, and $\Delta B_{x}$ represents the difference in the x-direction magnetic field at the two TMR locations, as illustrated in Figure 2c. Under small excitation and ambient magnetic fields, it can be assumed that $S_{R}B_{x}$ is much smaller than $R_{0}$, leading to a simplified differential output. First, since the two sets of TMR sensors are symmetrically arranged with respect to the excitation coil, they detect the same excitation magnetic field $B_{Coil}$. Second, given that the distance between the two TMR sets is on the order of centimeters, the environmental magnetic field $B_{Noise}$ can be considered identical for both. Additionally, when the probe is displaced in the lift-off direction, the lift-off distance $d_{lo}$ is the same for both TMR sets. If no defect is present, the secondary magnetic field $B_{Secondary}$ varies identically for both TMR sensors. In summary, the differential output of the proposed differential ACFM effectively eliminates the influence of the excitation and environmental magnetic fields while also canceling magnetic field variations caused by changes in lift-off distance in the absence of defects. As a result, this design reduces noise in the detection results and enhances sensitivity, which is further validated by subsequent experimental data.

### 2.2. Probe Design

**Figure 3:**
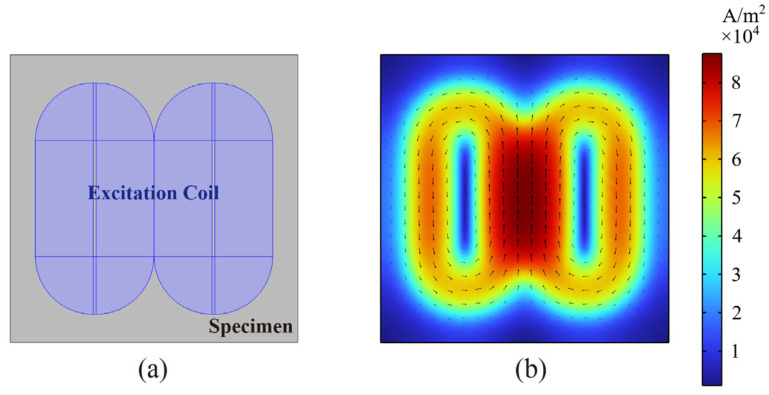
(**a**) Schematic of the finite element simulation model, the purple area is the coil; (**b**) current distribution on the surface of the simulated specimen.

A dual-racetrack planar coil was designed as the excitation unit for the differential ACFM probe. The overall dimensions of the coil are 50 mm × 45 mm, with a trace width of 0.254 mm, a spacing of 0.406 mm, and 20 turns per racetrack coil. To verify the effectiveness of the dual-racetrack planar coil, a finite element simulation model was established, as shown in Figure 3a. The simulated surface current distribution of the specimen is illustrated in Figure 3b, where a uniform induced current with the same direction is generated within the rectangular region at the center of the excitation coil. This ensures that the ACFM detection requirements are met.

**Figure 4:**
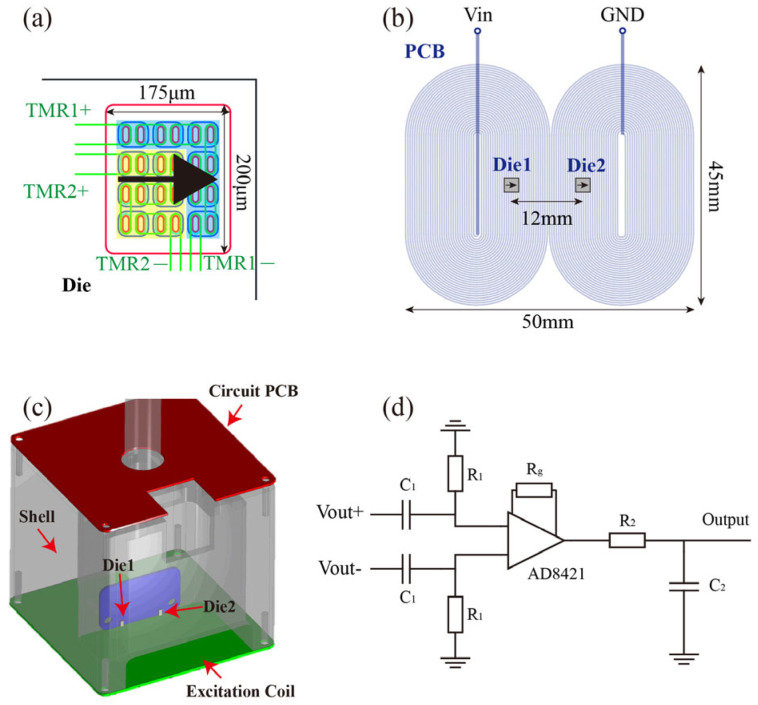
(**a**) TMR structure design; (**b**) relative position of the TMR chip and excitation coil; (**c**) schematic diagram of the probe structure; (**d**) schematic diagram of the interface circuit.

Additionally, a TMR chip was designed, as shown in Figure 4a. The chip integrates two TMR units in a 200 μm × 175 μm area, with each unit consisting of 12 series-connected MTJs. The TMR device is independently manufactured in the laboratory environment. The magnetoresistance ratio is 207%. The sensitivity near the zero field in the full-bridge configuration is 14.65 mV/V/Oe, and the detection limit is 0.27 nT/√Hz at 1 kHz.

During usage, the bare TMR chip is adhered to a PCB board, with electrode connections made via gold wires. The chip is then encapsulated using silicone for basic protection. The relative position of the TMR chip and the excitation coil is depicted in Figure 4b, where the TMR chips are 12 mm apart and positioned at the center of the coil. The complete probe structure is shown in Figure 4c. In addition to the core planar coil excitation unit and TMR sensing unit, the probe includes a 3D-printed outer casing and a sensor interface circuit PCB. The excitation coil is affixed to the bottom of the casing, while the TMR and its PCB lead-out board are mounted inside the casing near the coil using screws. The interface circuit PCB is secured to the upper part of the casing. The interface circuit, illustrated in Figure 4d, performs preliminary processing of the Wheatstone bridge output, including band-pass filtering in the 100–1000 Hz range and 10× signal amplification to enhance detection performance.

It is worth noting that conventional TMR sensors also utilize a Wheatstone bridge configuration to achieve optimal performance, which requires four TMR units. Therefore, our design does not increase the number of TMR sensors needed. Additionally, the excitation source and back-end signal processing in this probe remain the same as those in standard ACFM systems, eliminating the need for redesign. As a result, implementing this design in practical engineering applications does not incur any additional costs.

## 3. Experiments and Results

### 3.1. Experimental Setup

Based on the differential TMR-ACFM probe, a complete detection system was independently developed, with experimental tests performed on aluminum alloy surface defects. Analysis of the results demonstrates the design’s effectiveness in noise suppression and detection quality enhancement, highlighting its potential for high-precision nondestructive testing applications.

**Figure 5:**
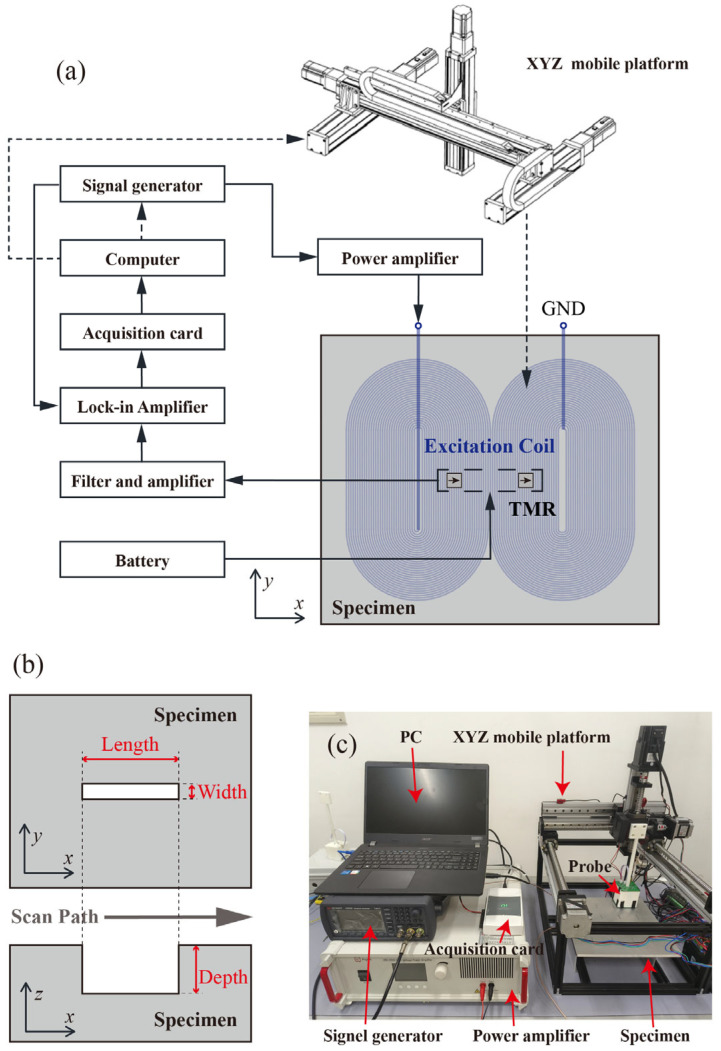
(**a**) Block diagram of the differential TMR-ACFM detection system; (**b**) defect specimen and scanning direction; (**c**) photograph of the detection system.

The differential TMR-ACFM detection system, as shown in Figure 5a, consists of excitation, probe, signal processing and acquisition, and motion control. An alternating signal generated by a signal generator is amplified tenfold by a power amplifier before being applied to the excitation coil. The Keysight 33510B signal generator in use supports arbitrary waveform generation up to 20 MHz with a peak-to-peak voltage of up to 10 Vpp. To eliminate power line noise interference, the differential ACFM probe is powered by a 5 V dry battery. The probe’s differential output is amplified and filtered before entering a lock-in amplifier, which uses the reference signal from the signal generator to extract the in-phase component. The lock-in amplifier is the SR860 of SRS company and can detect voltage signals with peaks below 1 V within the range of 1 MHz to 500 kHz. The real part of the signal is then acquired using an NI-6210 data acquisition card and transmitted via USB to a computer for recording. The resolution of the acquisition card is 16 bits, and the maximum sampling rate is 250 kS/s. During testing, the sample remains stationary while the probe, mounted on a computer-controlled XYZ mobile platform, scans for defects. The customized platform achieves a repetitive positioning accuracy of 0.05 mm. The specimen is an 8 mm-thick 6061 aluminum alloy plate with artificial cracks fabricated using electrical discharge machining. A total of 13 crack sizes were set, as listed in Table 1, and their layout, along with the probe scanning direction, is shown in Figure 5b. A LabVIEW program was developed to synchronize motion control and signal acquisition. During experiments, the probe scanning speed was set to 10 mm/s, with a sampling frequency of 100 Hz. Each scan covered 50 mm, yielding a signal sequence of 500 data points per scan.

### 3.2. Results Analysis

In the evaluation of ACFM probe performance, the quality factor P is commonly used to quantify the probe’s detection capability. Its formula is given by the following:

(4) $$P=\frac{max-min}{ref}\times 100\%,$$

where max represents the peak value of the signal fluctuation caused by the defect, min represents the valley value of the signal fluctuation caused by the defect, and ref is the reference signal value in the absence of defects. The unit of P is expressed as a percentage (%). The reference value ref is obtained by averaging the signal at the edges of the detection region, where the output is minimally affected by the crack due to the greater distance from the defect.

In addition to P, the signal-to-noise ratio (SNR) is commonly used to quantify the quality of the probe’s output signal. The formula for SNR is given by:

(5) $$SNR=10lg\frac{max-min}{noise}$$

where noise refers to the signal fluctuation noise in the absence of defects, which is quantified as the standard deviation of the signal at the edges of the detection region. The unit of SNR is expressed in decibels (dB).

**Figure 6:**
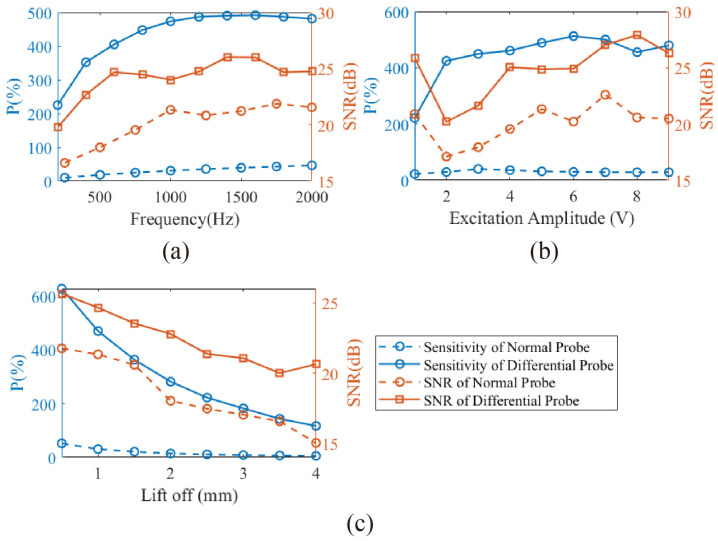
The relationships between the quality factor and signal-to-noise ratio (SNR) of the differential probe and conventional probe when detecting Crack #1 are analyzed concerning (**a**) frequency, (**b**) excitation voltage, and (**c**) lift-off distance.

To ensure that the excitation frequency, excitation voltage, and lift-off distance used in the experiments are within the optimal range, the three parameters were independently adjusted during the detection of Crack #1, and their corresponding quality factors P and SNR were calculated. The baseline parameters used in the experiments were 1 kHz, 5 V, and 1 mm. As shown in Figure 6a–c, the quality factor P of the differential probe initially increases with excitation frequency and excitation voltage before stabilizing, with turning points at 1 kHz and 2 V, respectively. Meanwhile, P decreases as the lift-off distance increases. Similarly, the SNR increases with excitation frequency and excitation voltage before stabilizing, with turning points at 750 Hz and 4 V, and decreases with increasing lift-off distance. Notably, at an excitation voltage of 1 V, the probe’s output SNR exhibits an anomalous increase, which is attributed to the signal generator switching its range, introducing less noise under a lower range setting. Overall, the chosen parameters of 1 kHz, 5 V and 1 mm fall within the optimal range for both P and SNR, making them suitable for subsequent experiments. Additionally, the same tests were conducted on a conventional ACFM probe, and the results show that under all parameter combinations, the signal quality of the differential module consistently outperforms that of the conventional probe. Specifically, the quality factor P is improved by more than an order of magnitude, and the SNR is generally enhanced by over 3 dB. These experimental results confirm that the differential probe design effectively suppresses noise while enhancing detection capability.

**Figure 7:**
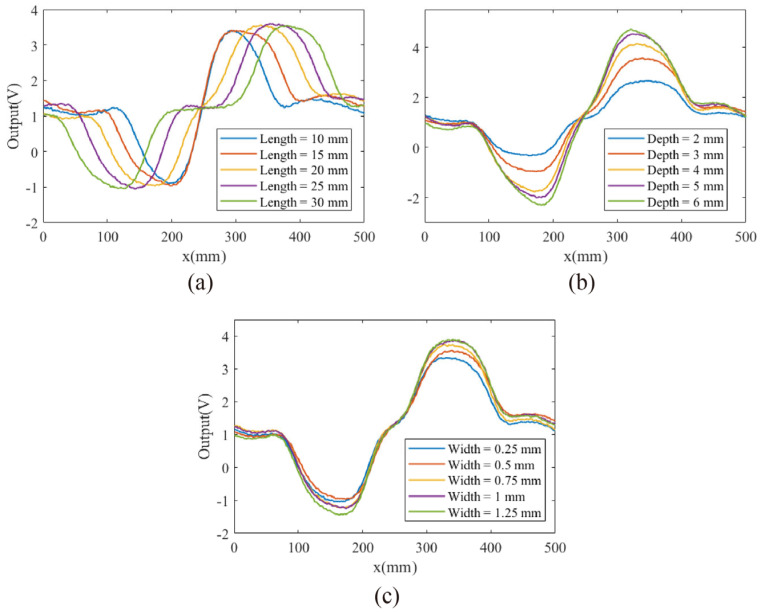
The output signals of the differential probe were analyzed when detecting cracks with varying (**a**) lengths, (**b**) depths, and (**c**) widths.

After establishing the experimental parameters, 13 distinct defects were tested using the differential probe to investigate the impact of variations in defect size on the output signal. Defects #1, #10, #11, #12, and #13 differ only in length, as shown in Figure 7a. The results indicate that defect length significantly affects the peak and valley positions of the output signal but has little impact on signal intensity. Defects #1, #2, #3, #4, and #5 differ only in depth, as shown in Figure 7b, where defect depth has a minimal effect on peak and valley positions but strongly influences signal intensity. Defects #1, #6, #7, #8, and #9 differ only in width, as shown in Figure 7c, revealing that defect width has a relatively minor effect on both the peak–valley positions and signal intensity. These results suggest that defect size influences the shape of the output signal curve, making it possible to directly infer defect dimensions from the signal, thus enabling quantitative defect measurement.

## 4. Crack Evaluation Based on Deep Learning

### 4.1. Dataset

The key to crack evaluation lies in accurately determining crack dimensions from the output signal, which mathematically constitutes an inverse problem. Given the complex physical variations in ACFM signals, many intermediate processes exhibit nonlinearity. Deep learning, being highly effective in solving complex problems, provides a promising approach to address this challenge. Compared to traditional machine learning methods [21], deep learning demonstrates significant advantages through its automated feature learning mechanism, which enables superior generalization capabilities and enhanced capacity for handling complex datasets with intricate nonlinear relationships.

It is proposed to design a deep learning model for crack size inversion. The network takes the signal curve obtained from the differential ACFM system as input and outputs three numerical values representing crack length, depth, and width. By leveraging deep neural networks, the model can learn the intricate relationships between the defect-induced signal variations and crack dimensions, overcoming the limitations of conventional analytical methods.

Building a dataset is the foundation for training deep learning models. Using the previously mentioned differential ACFM system, 30 measurements were conducted for each of the 13 cracks, resulting in a total of 390 data samples. Each sample consists of a one-dimensional sequence with a length of 500. For subsequent experiments, the dataset was divided into three parts: 80% for the training set, 10% for the validation set, and 10% for the test set. This partitioning ensures effective model training, tuning, and evaluation, helping to improve generalization performance in crack size estimation.

To address the issue of limited data, this study utilizes data augmentation techniques to enhance the model’s generalization ability. Specifically, the methods involve introducing random translations within ±5 mm of the original samples to simulate the uncertainty in crack positions. Additionally, up to 5% random noise relative to the maximum signal value is applied. This approach aims to create a diversified training dataset, effectively mitigating overfitting risks and strengthening the model’s robustness, thereby ensuring reliability for industrial applications.

### 4.2. Model Architecture and Training

From the previous experimental results, it is observed that defect size correlates with both signal intensity and peak–valley positions. The former can be considered a local feature, while the latter represents a global feature. To accurately capture both aspects, our evaluation model should be capable of learning both local and global features effectively. Among common deep learning models for processing 1D sequential signals, CNNs excel at learning local features [22], whereas Transformers are more suited for capturing global dependencies [23]. A straightforward approach is to combine CNN and Transformer networks; however, in practice, this may not yield optimal results. The complexity of the Transformer model is more suitable for extremely large datasets and highly complex problems. Given the limitations of our experimental environment and the relatively small dataset, a full Transformer model may not be the best choice. Since the core advantage of Transformers lies in their attention mechanism, the proposal involves replacing it with a lightweight attention module to retain the ability to capture global dependencies while reducing computational overhead. This approach allows us to enhance the performance of our deep learning model while ensuring it remains efficient and suitable for our dataset size.

**Figure 8:**
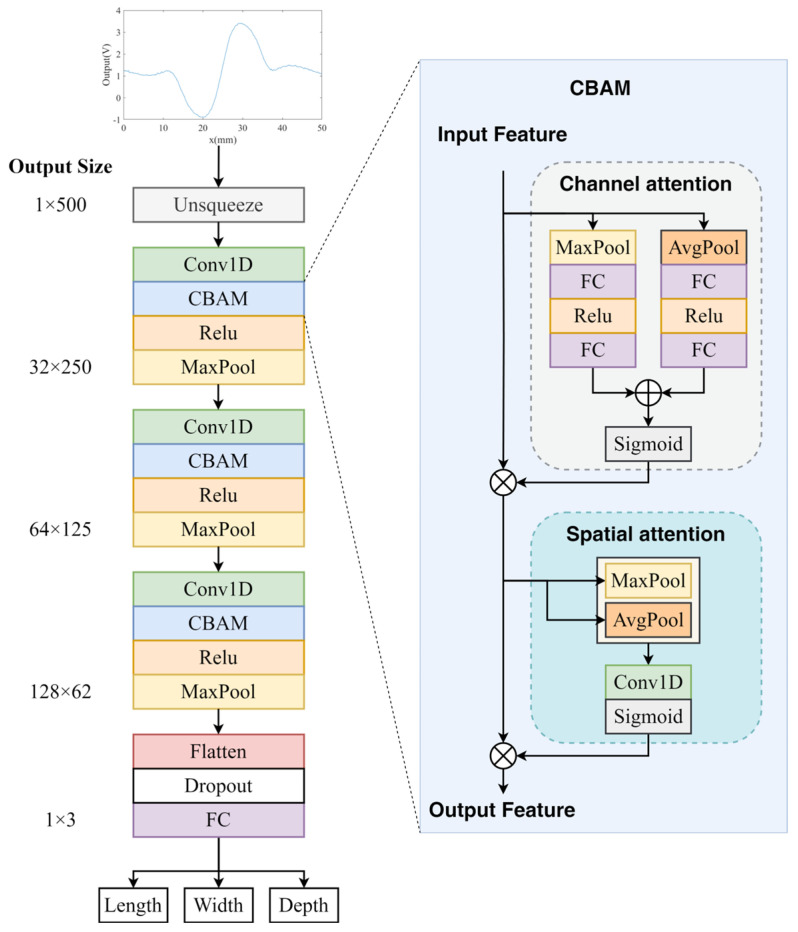
CNN + CBAM model architecture.

Based on this approach, the CNN + CBAM network was constructed, as shown in Figure 8. The 1D input data are first processed through an Unsqueeze layer to expand the channel dimension. The features then pass sequentially through a Conv1D layer, a CBAM module, a ReLU activation layer, and a MaxPool layer. After repeating this process three times, the features are flattened and passed through a Dropout layer (Dropout rate of 0.3) and fully connected layers to output three defect size parameters. Dropout, as a classical regularization technique, simulates ensemble training by randomly deactivating neurons, effectively enhancing the model’s generalization ability. In this architecture, the Conv1D layers use a kernel size of 3, while the pooling layers use a kernel size of 2. The number of channels expands to 32, 64, and 128 through the three Conv1D layers. The key component of the model is the CBAM (Convolutional Block Attention Module), which consists of two submodules [24]: the Channel Attention Module (CAM) and the Spatial Attention Module (SAM). CAM applies max pooling and average pooling along the spatial dimension, sums the outputs, and activates them through a Sigmoid layer to generate channel-wise attention weights. This mechanism allows the model to focus on more informative channels. Similarly, SAM performs max pooling and average pooling along the channel dimension, concatenates the outputs, and processes them through a convolutional layer followed by a Sigmoid activation to generate spatial attention weights. This helps the model focus on the most informative spatial regions. Since both submodules incorporate global pooling, the model effectively enhances its ability to capture global information while maintaining the local feature extraction capability of CNNs.

CNN and its multimodal fusion models have been widely applied in industrial inspection [25,26,27]. The CNN-CBAM model proposed in this study achieves autonomous focus on key features through dual-channel attention mechanisms, with its core advantage lying in dynamically adaptive feature selection. This method can further expand into engineering scenarios such as precision manufacturing inspection, building health monitoring, geological disaster warning, and physiological signal analysis.

**Figure 9:**
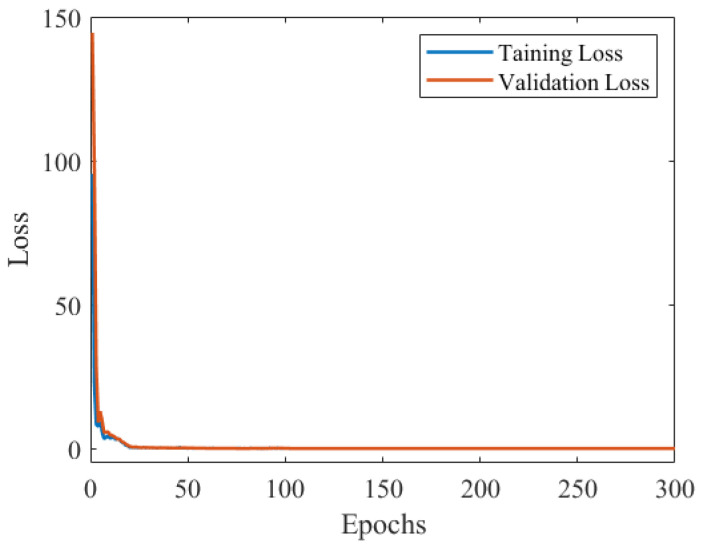
The loss curve of model training.

Since defect size evaluation is essentially a regression problem, the mean squared error (MSE) is used as the loss function. The Adam optimizer is chosen for its ability to adaptively adjust the learning rate while requiring less memory. Key hyperparameters optimized via the PSO algorithm include batch size, learning rate, and reduction ratio for CBAM. Parameter ranges were set as learning rate ($1\times 10^{-5}$ to 0.1), batch size (16 to 128), and reduction ratio (8 to 64). The optimization process spanned 300 epochs, optimizing against the validation set average error. Using 10 particles over 50 iterations, the optimal parameters were determined as learning rate 8.94 × 10^−3^, batch size 72, and reduction ratio 8, which were used for training. The loss curve of training is shown in Figure 9. The loss curves for the training and validation sets converge with almost no visible gap after stabilization, indicating that the model achieves a perfect fit.

### 4.3. Results and Discussion

To validate the advantages of the proposed model, additional comparative models were constructed, including a CNN model, a Transformer model, and a CNN + Transformer model. The CNN and Transformer models each consisted of three layers, while the CNN + Transformer model was designed with alternating convolutional and Transformer layers, each with three layers, making its structure similar to the CNN + CBAM model used in this study. All models employ PSO for hyperparameter optimization strategy, followed by training for 300 epochs.

The prediction errors for crack size estimation were evaluated on the test set, with the average relative errors summarized in Table 2. The results show that, compared to the Transformer model, the CNN model exhibited larger errors in length estimation but smaller errors in depth and width estimation. This confirms that CNN is more effective in learning local features, whereas the Transformer model is better suited for capturing global features, aligning with the previous analysis. The CNN + Transformer model demonstrated length errors similar to those of the Transformer model, while its depth and width errors were intermediate between those of the CNN and Transformer models. This indicates that the hybrid model did not fully inherit the CNN’s capability for local feature extraction. In contrast, the CNN + CBAM model achieved the lowest prediction errors across all three crack size parameters, demonstrating its superior performance in crack size estimation. Specifically, the CNN + CBAM model exhibited lower length errors compared to the CNN + Transformer model, indicating that the CBAM module in this study provided stronger global feature extraction capabilities than the Transformer layers. Additionally, the CNN + CBAM model achieved lower depth and width errors than the CNN model, suggesting that the introduction of channel and spatial attention mechanisms enhanced its ability to capture local features. These findings highlight the effectiveness of the CNN + CBAM model in crack size inversion, offering a more accurate approach for estimating crack length, depth, and width. Notably, the depth error is significantly larger than the length and width errors. This is because, in ACFM, the width has a minimal impact on the current distribution, resulting in only slight variations in the output curve.

For comprehensive model performance assessment, a multidimensional statistical metrics system was constructed, as shown in Table 3, with key metrics computed for all models based on the test dataset, as shown in Table 4. The results indicate that the CNN + CBAM model outperforms other comparative models across critical metrics. This reflects the model’s comprehensive advantages in average prediction accuracy, systematic bias control, and extreme error suppression.

**Figure 10:**
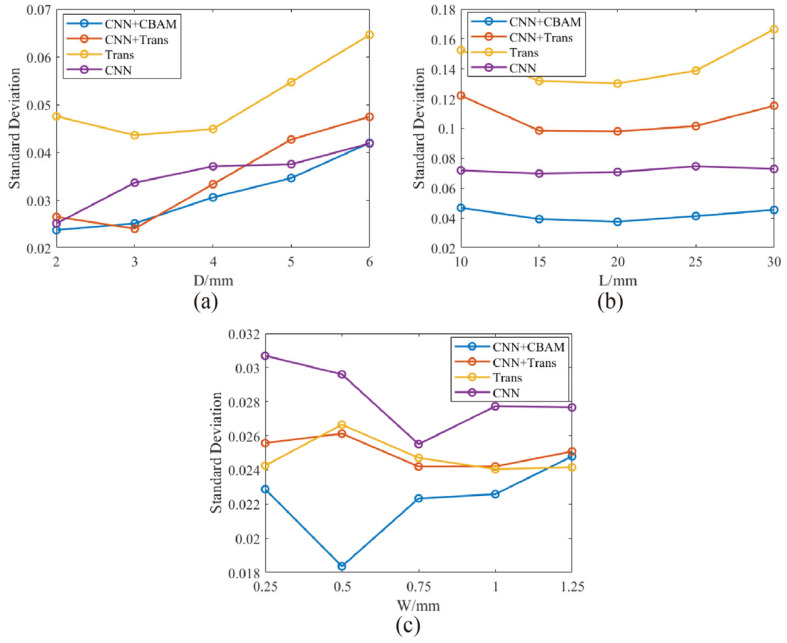
The relationship between estimated standard deviation and true values in predicting crack (**a**) length, (**b**) depth, and (**c**) width.

To evaluate prediction uncertainty, the Monte Carlo Dropout method was employed. During testing, Dropout remained active, and each test sample underwent 50 independent forward passes. Statistical distribution characteristics of prediction results were collected to compute prediction standard deviations, offering insights into the model’s confidence levels across different samples (Figure 10). This approach effectively quantified prediction uncertainties, facilitating reliable decision-making in complex industrial detection scenarios.

**Figure 11:**
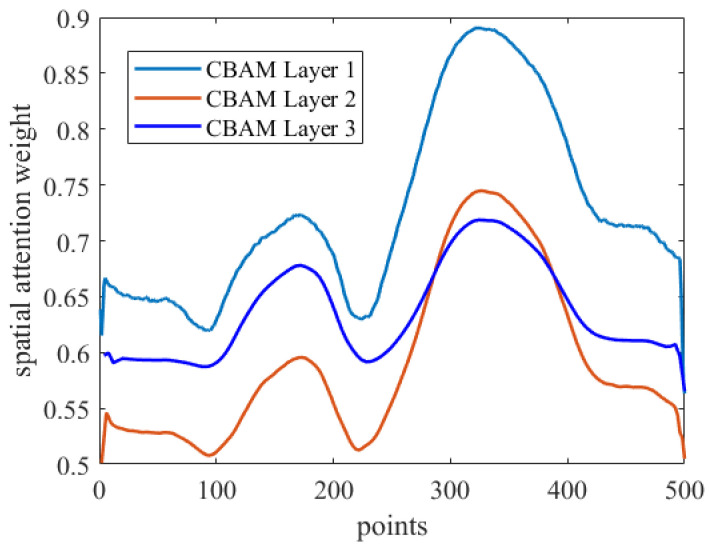
Correspondence between the spatial attention weights of the three CBAM layers and the original sequence.

Given the inherent complexity of deep learning models, interpretability analysis was crucial. This study utilized attention mechanisms to interpret feature prioritization, where weights indicated the model’s focus on specific features. Figure 11 illustrates the relationship between spatial attention weights in the three CBAM layers and the original input sequence, showcasing how CBAM enhanced model attention towards critical features like peak and valley positions in the input curve, crucial for defect size detection.

In conclusion, the CNN + CBAM network integrated with differential ACFM demonstrated high accuracy in predicting crack dimensions, validating the effectiveness of the proposed approach and highlighting the potential of differential ACFM for quantitative crack detection in industrial applications.

## 5. Conclusions

This study developed a differential TMR-ACFM probe combined with a CNN + CBAM network for quantitative assessment of crack sizes. The conclusions of this study are as follows:

(1) Theoretical and experimental evidence demonstrates that the differential probe design effectively suppresses noise introduced by lift-off effects and external electromagnetic interference, significantly enhancing detection sensitivity. Utilizing a differential TMR bridge as the sensitive unit, the probe does not increase manufacturing costs compared to traditional ACFM probes, with the quality factor of the differential signal improving by over tenfold and the signal-to-noise ratio increasing by more than 3 dB.
(2) The shape of the output signal from the differential probe correlates with the length, depth, and width of cracks. Length primarily relates to global features of the output curve, while width and depth are mainly associated with local features.
(3) Based on experimental data from the differential probe, the designed CNN + CBAM network effectively predicts crack dimensions. For cracks with lengths of 10–30 mm, depths of 2–6 mm, and widths of 0.25–1.25 mm, the relative prediction errors are 0.201% (length), 0.709% (depth), and 7.224% (width), respectively, demonstrating superior accuracy compared to several other deep learning architectures.

In future research endeavors, the differential probe will be utilized for acquiring magnetic field maps and integrating deep learning techniques to enable three-dimensional defect reconstruction. Additionally, the incorporation of phase information is planned to enhance the detection capability for internal defects.

## Figures and Tables

**Table 1:** The crack size on the specimen.

Number	Length/mm	Depth/mm	Width/mm
#1	20	3	0.5
#2	20	2	0.5
#3	20	4	0.5
#4	20	5	0.5
#5	20	6	0.5
#6	20	3	0.25
#7	20	3	0.75
#8	20	3	1
#9	20	3	1.25
#10	10	3	0.5
#11	15	3	0.5
#12	25	3	0.5
#13	30	3	0.5

**Table 2:** The relative error of four models in predicting crack size.

Model	Length Error (%)	Depth Error (%)	Width Error (%)
CNN	1.217	0.899	10.21
Transformer	0.580	1.768	13.94
CNN + Transformer	0.858	0.986	10.58
CNN + CBAM	0.201	0.709	7.224

**Table 3:** Statistical metrics to evaluate the network models [21].

Indicator	Formula
Coefficient of determination	$R^{2}=1-\frac{\sum_{i=1}^{n}{\left(Actual_{i}-Predicted_{i}\right)}^{2}}{\sum_{i=1}^{n}{\left(Actual_{i}-Actual_{\mathrm{avg}}\right)}^{2}}$
Mean squared error	$MSE=\frac{1}{n}\sum_{i=1}^{n}{\left(Actual_{i}-Predicted_{i}\right)}^{2}$
Mean absolute error	$MAE=\frac{1}{n}\sum_{i=1}^{n}|Actual_{i}-Predicted_{i}|$
Mean absolute relative error	$MARE=\frac{1}{n}\sum_{i=1}^{n}\frac{\left|Actual_{i}-Predicted_{i}\right|}{Actual_{i}}$
Mean square relative error	$MSRE=\frac{1}{n}\sum_{i=1}^{n}\frac{{\left(Actual_{i}-Predicted_{i}\right)}^{2}}{Actual_{i}}$
Mean bias error	$MBE=\frac{1}{n}\sum_{i=1}^{n}\left(Actual_{i}-Predicted_{i}\right)$
Maximum absolute relative error	$erMAX=\max\left(\frac{\left|Actual_{i}-Predicted_{i}\right|}{Actual_{i}}\right)$

**Table 4:** Performance metrics for four models.

**Model**	$R^{2}$	MSE	MAE	MARE	MSRE	MBE	erMAX
CNN	0.9981	0.0111	0.0887	0.0551	0.0114	−0.0600	0.4139
Transformer	0.9974	0.0134	0.1065	0.0975	0.0157	−0.0049	0.3063
CNN + Transformer	0.9983	0.0123	0.0917	0.0808	0.0131	0.0326	0.3423
CNN + CBAM	0.9993	0.0078	0.0688	0.0434	0.0081	−0.0082	0.1407

## Data Availability

The data presented in this study are available on request from the corresponding author because more related work is still ongoing.

## Abbreviations

The following abbreviations are used in this manuscript:

ACFM	Alternating current field measurement
NDT	Nondestructive testing
GMR	Giant magnetoresistance
TMR	Tunnel magnetoresistance
CNN	Convolutional Neural Network
CBAM	Convolutional Block Attention Module
SNR	Signal-to-noise ratio
CAM	Channel Attention Module
SAM	Spatial Attention Module
MSE	Mean squared error
PSO	Particle Swarm Optimization
